# Antibody-drug conjugate-related marker heterogeneity between primary tumors and metastatic lymph nodes in advanced urothelial cancers

**DOI:** 10.1515/jtim-2026-0039

**Published:** 2026-06-13

**Authors:** Xingliang Tan, Neng Jiang, Juehui Li, Haihong Yang, Xinpei Deng, Chichen Zhang, Qianghua Zhou, Zhicheng Liu, Runhao Zheng, Jiamin Zeng, Shengjie Guo, Zhiming Wu, Kai Yao, Yanjun Wang

**Affiliations:** Department of Urology, Sun Yat-sen University Cancer Center, Guangzhou, Guangdong Province, China; State Key Laboratory of Oncology in Southern China, Guangzhou, Guangdong Province, China; Guangdong Provincial Clinical Research Center for Cancer, Guangzhou, Guangdong Province, China; Department of Pathology, Sun Yat-sen University Cancer Center, Guangzhou, Guangdong Province, China; Department of Neurosurgery, Sun Yat-sen University Cancer Center, Guangzhou, Guangdong Province, China; Department of Urology, Gansu Hospital, Affiliated Cancer Hospital of Sun Yat-Sen University, Lanzhou, Gansu Province, China

## To the editor

Urothelial carcinoma (UC) also known as transitional cell carcinoma which encompasses carcinomas of the renal pelvis, ureter, bladder and urethra, is a common genitourinary malignancy worldwide. About 5%–15% of UC patients have local advanced or metastatic disease (la/mUC) and suffer from an unfavorable prognosis.^[1]^ Although traditional cisplatin-based chemotherapy has served as the standard treatment for la/mUC for decades, up to 50% of individuals are ineligible for cisplatin and are limited by severe hematological toxicity.^[2]^ Fortunately, antibody-drug conjugates (ADCs) such as HER2, HER3, Nectin4 and Trop2-targeted therapies offer a promising, effective and low-toxicity innovative strategy and have significantly increased clinical outcomes and quality of life in UC.^[3, 4, 5, 6, 7]^ Despite the remarkable progress facilitated by ADC-targeted therapy, previous studies lacked a systematic, large sample size, Asian population-based standardized scoring system to detect ADC-related gene expression and failed to explore the expression heterogeneity of primary tumors (PTs) and matched metastatic lymph nodes (mLNs) in advanced disease. A thorough investigation of ADC gene expression in mLNs is particularly important in the perioperative decision-making process of la/mUC patients, and it would provide prognostic value for treatment and follow-up.

**Figure 1:**
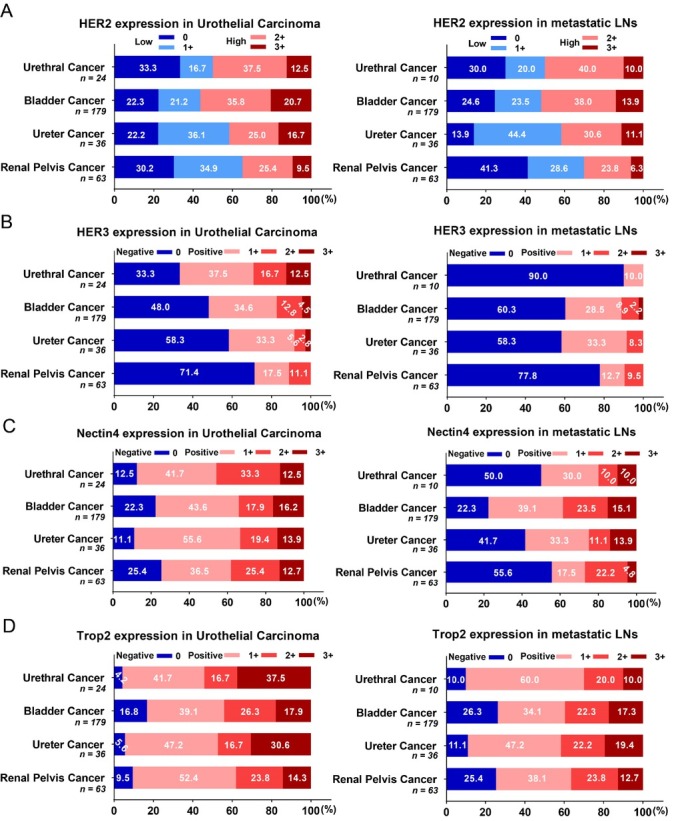
The expression of ADC-relative targets in paired tissues of urothelial cancers. ADC: antibody-drug conjugate. (A)HER2 immunohistochemical staining score of 0/1+ was identified as HER2 low expression and 2/3+ was HER2 high expression. (B-D) Immunohistochemical staining score of 0 was regarded as negative expression in HER3, Nectin4 and Trop2. ADC: antibody-drug conjugate; LNs: lymph nodes.

In this study, 302 drug-naïve advanced UC patients, including 179 with bladder cancer, 63 with renal pelvis cancer, 36 with ureter cancer, and 24 with urethral cancer were detected to explore the expression patterns and heterogeneity of HER2, HER3, Nectin4 and Trop2 in paired tissues. Patient selection, baseline information and the methods and scoring criteria and expression pattern of immunohistochemistry (IHC) are described briefly in Supplementary Table 1 and Figures 1–3. All IHC slides were independently evaluated and scored by two experienced senior pathologists in a double-blind manner.

With respect to primary tumors (PT), 75.2% of advanced UC patients had positive HER2 expression (1–3+), of which 49.7% had higher HER2 expression (2/3+). The HER3-, Nectin4- and Trop2-positive tumor rates were 46.4%, 79.1% and 87.1%, respectively (Figure 1). However, the positive rates were reduced in mLNs, with only 72.9% for HER2 expression (45.8% high expression), 35.4% for HER3, 67.0% for Nectin4 and 76.4% for Trop2. When comparing different primary sites, we found that HER2 overexpression (56.5%) was more common in bladder cancer, whereas overexpression rates were lower in upper tract urothelial carcinomas (UTUCs), with a 34.9% HER2 overexpression rate in the renal pelvis and a 41.7% HER2 overexpression rate in ureter cancer. A similar trend was also found in matched mLNs, where there was a HER2 overexpression rate of 52.0% in bladder metastatic nodes but only 30.2% and 41.7% in pN+ renal pelvis and ureter metastatic nodes, respectively (Figure 1). In contrast, positive Trop2 (renal pelvis: 90.5%, ureter: 94.4%, bladder: 79.8%) and Nectin4 (renal pelvis: 74.6%, ureter: 88.9%, bladder: 77.7%) expression in tumors was more frequent in UTUCs than in bladder cancer. The results indicated that the primary sites of the genitourinary tract result in diverse target gene expression preferences, which could also lead to different choices of ADC-targeting therapeutic strategies and outcomes.

Subsequently, survival analysis revealed that high HER2 expression (2/3+) and positive HER3 and Trop2 expression (1–3+) were associated with shorter OS and PFS in patients with advanced UC (Supplementary Tables 2–5 and Figure 4). However, only HER2 overexpression in mLNs was an independent prognostic ADC-related gene factor for predicting poor OS (HR = 2.20; 95% CI, 1.43–3.37; *P* < 0.001) and PFS (HR = 1.62; 95% CI, 1.07–2.45; *P* = 0.022). Our results suggest the significant prognostic implications of these markers in tumors, especially in mLNs, which deserve more attention.

More importantly, there is striking heterogeneity in biomarker expression between PTs and mLNs in advanced UC patients (Supplementary Figure 5). HER2 and HER3 had the highest concordance rate (71.9% in advanced UC) between primary tumors and metastatic lymph nodes, while the concordance rates for Trop2 and Nectin4 were 65.3% and 50.7%. In addition, the heterogeneous expression of target genes appeared to have little association with the location of the primary tumor site. For example, the concordance rate of HER2 expression in paired tissues was 66.6% in renal pelvic cancer, 75.0% in ureteral cancer, 73.2% in bladder cancer and 70% in pN+ ureter cancer. Similarly, the HER3 expression concordance rates were 84.1%, 77.8%, 66.5% and 70.0%; the Nectin4 expression concordance rates were 36.5%, 41.7%, 57.0% and 60.0%; and the Trop2 expression concordance rates were 65.1%, 63.9%, 64.2% and 90.0%, respectively. We found that patients with low HER2 expression in PTs but high HER2 expression in mLNs experienced the shortest mOS at only 15.13 months, compared with 25.93 months for patients with consistent HER2 expression, and those with high-HER2 expression in PTs but low-HER2 expression in mLNs had the best mOS of approximately 68.03 months (*P* = 0.033). These results suggest that elevated HER2 expression in mLNs indicates the aggressive potential of tumor progression associated with poor survival in advanced UC patients. Besides, it emphasizes the necessity of assessing gene expression in both primary and metastatic tissues to accurately predict patient outcomes. Although HER3, Trop2 and Nectin4 heterogeneous expression are not associated with prognosis, we still recommend identifying the expression patterns to develop a more personalized and effective ADCs-targeted strategy, which might ultimately improve the survival of patients with advanced UC.

We conclude that advanced UC undergoes extensive dynamic evolution during progression, including the heterogeneous expression of ADC-related markers, which could be the underlying reason for resistance to ADC-targeted therapy. We detected significant heterogeneity in expression between paired tissues, with concordance rates ranging from 50.7% (Nectin4) to 71.9% (HER2 and HER3). Downregulation of markers in mLNs was far more common than upregulation—most notably for Nectin4 (29.9% downregulation) and Trop2 (22.6% downregulation) —suggesting that tumor progression to lymph nodes is often accompanied by loss of ADC target expression. These findings suggest that relying solely on PT Nectin4 status could misclassify nearly half of advanced UC patients, potentially depriving those with mLN-specific Nectin4 expression of effective therapy. Similarly, Ghali *et al*.^[8]^ reported stable Trop2 H-scores between PTs and distant metastases in patients with UC, but our study extends this insight by demonstrating Trop2 downregulation in mLNs. In addition, another critical clinical insight from our study is the prognostic significance of mLN HER2 expression. Multivariate Cox regression analysis revealed HER2 overexpression in mLNs as the only independent ADC-related prognostic factor for poor OS and PFS. Notably, patients with low-HER2 expression in PTs but high-HER2 expression in mLNs had the shortest median OS, compared to 25.93 months for patients with concordant HER2 expression and 68.03 months for those with high-HER2 PT but low-HER2 mLN expression. These findings underscore the limitations of relying solely on primary tumor biopsies for prognostic stratification; in our cohort, 9.7% of patients had elevated HER2 expression in mLNs relative to PTs, and these patients would have been missed by primary-only testing—potentially depriving them of HER2-targeted ADCs that could improve outcomes.

We also observed site-specific differences in marker expression that may guide ADC selection. UTUC patients may be better candidates for Trop2-targeted ADCs or Nectin4-targeted ADCs, whereas bladder cancer patients may derive greater benefit from HER2-targeted agents. This site specificity has not been previously reported in ADC marker studies and could inform subtype-specific clinical trials—an important consideration given that UTUCs and bladder cancer often have distinct molecular profiles and clinical outcomes. Therefore, detecting gene expression in both primary and metastatic tissues is recommended for clinical decision-making to enhance the effectiveness of ADCs therapies, especially in advanced UC patients.

## Supplementary Material

Supplementary Material Details

## References

[1] Bray F, Laversanne M, Sung H, Ferlay J, Siegel RL, Soerjomataram I (2024). Global cancer statistics 2022: GLOBOCAN estimates of incidence and mortality worldwide for 36 cancers in 185 countries. CA Cancer J Clin.

[2] Bellmunt J, von der Maase H, Mead GM, Skoneczna I, De Santis M, Daugaard G (2012). Randomized phase III study comparing paclitaxel/cisplatin/gemcitabine and gemcitabine/cisplatin in patients with locally advanced or metastatic urothelial cancer without prior systemic therapy: EORTC Intergroup Study 30987. J Clin Oncol.

[3] Powles T, Rosenberg JE, Sonpavde GP, Loriot Y, Durán I, Lee JL (2021). Enfortumab Vedotin in Previously Treated Advanced Urothelial Carcinoma. N Engl J Med.

[4] Powles T, Valderrama BP, Gupta S, Bedke J, Kikuchi E, Hoffman-Censits J (2024). Enfortumab Vedotin and Pembrolizumab in Untreated Advanced Urothelial Cancer. N Engl J Med.

[5] Sheng X, Wang L, He Z, Shi Y, Luo H, Han W (2024). Efficacy and Safety of Disitamab Vedotin in Patients With Human Epidermal Growth Factor Receptor 2-Positive Locally Advanced or Metastatic Urothelial Carcinoma: A Combined Analysis of Two Phase II Clinical Trials. J Clin Oncol.

[6] Tagawa ST, Balar AV, Petrylak DP, Kalebasty AR, Loriot Y, Fléchon A (2021). TROPHY-U-01: A Phase II Open-Label Study of Sacituzumab Govitecan in Patients With Metastatic Urothelial Carcinoma Progressing After Platinum-Based Chemotherapy and Checkpoint Inhibitors. J Clin Oncol.

[7] Weng W, Meng T, Pu J, Ma L, Shen Y, Wang Z (2023). AMT-562, a Novel HER3-targeting Antibody-Drug Conjugate, Demonstrates a Potential to Broaden Therapeutic Opportunities for HER3-expressing Tumors. Mol Cancer Ther.

[8] Ghali F, Vakar-Lopez F, Roudier MP, Garcia J, Arora S, Cheng HH (2023). Metastatic Bladder Cancer Expression and Subcellular Localization of Nectin-4 and Trop-2 in Variant Histology: A Rapid Autopsy Study. Clin Genitourin Cancer.

