# Supplementary material for: Antibody-drug conjugate-related marker heterogeneity between primary tumors and metastatic lymph nodes in advanced urothelial cancers
Source: J Transl Int Med. 2026 Jun 13;14(3):488–91. doi: 10.1515/jtim-2026-0039 (PMC13320528; doi:10.1515/jtim-2026-0039)
Supplement: Supplementary file 1 — Supplementary Material Details [file jtim-2026-0039_sm.pdf]

Supplementary materials

ADC-related marker heterogeneity between primary tumors and metastatic lymph nodes in advanced urothelial cancers

Patients cohort

This retrospective study enrolled advanced UC patients at Sun Yat-sen University Cancer Center (SYSUCC) from January 2008 to December 2023. Patient eligibility criteria included (1) undergoing radical surgery with pathologically confirmed UC and (2) detailed clinical and survival data and available tumor sections for IHC staining. Pathology information was individually determined by experienced pathologists at SYSUCC on the basis of the 8th edition of the AJCC TNM Staging System. The exclusion criteria were insufficient tumor tissue, poor tissue fixation, and the absence of clinicopathologic features. This study was performed in accordance with the Declaration of Helsinki and received approval from the ethics committee of Sun Yat-sen University Cancer Center (B2024-058-01), and informed consent was waived by the institutional review board due to the retrospective nature of the investigation.

Assessment of HER2, HER3, Nectin4 and Trop2 status

In brief, 4-μm paraffin-embedded, well-preserved tumor sections from both primary and metastatic lymph nodes were retrieved for regular pathological examination by hematoxylin and eosin (HE) staining. The expression of HER2, HER3, Nectin4 and Trop2 in patients involved in this study was evaluated by IHC staining. HER2 expression was automatically detected by an anti-HER2/neu (4B5) rabbit monoclonal antibody (Ventana; Roche Diagnostics) on the Ventana Bench Mark XT platform. All procedures were set and reproducible on the basis of the Ventana platform and represented the gold standard for anti-HER2 therapies[1]. The scoring criteria were based on the consensus of the Expert Committee on Urothelial Carcinoma of the Chinese Society of Clinical Oncology in 2021[2]. HER2 2+ or 3+ was considered to indicate high HER2 expression as previously described[3]. HER3, Nectin4 and Trop2 were evaluated by H-scores (range 0-300, rank 0 to 3+) multiplied by the extent and intensity of the staining[4-6]. H-scores >15 were considered to indicate positive expression (>15 and ≤100 was 1+, >100 and ≤200 was 2+, and >200 and ≤300 was 3+). The antibodies used were as follows: anti-HER3 (Cell Signaling Technology, HER3/ErbB3, #12708), anti-Nectin4 (Abcam, ab192033), and anti-Trop2 (Signaling Technology, TACSTD2/TROP2, #76730). The scoring was performed by two pathologists (JN and CKM) who were blinded to the clinical data. If

the results were inconsistent, the slides were reexamined, and a consensus was reached after discussion.

Statistical analysis

Student's or Wilcoxon tests were used for comparisons between two groups, while chi-square or Fisher's exact tests were used for qualitative variables. Sankey diagrams were constructed to summarize HER2, HER3, Nectin4 and Trop2 status evolution from the primary tumor to the matched metastasis. The independent predictors of HER2 discordance were identified using univariate and multivariate Cox regression analyses. Odds ratios (ORs) were used to measure the risks associated with various factors contributing to HER2 discordance with 95% CIs. Progression-free survival (PFS) and overall survival (OS) were analyzed using the Kaplan–Meier method and the log-rank test. All tests were two-sided, and statistical significance was set at $P<0.05$.

Patient Characteristics

A total of 4525 patients referred to SYSUCC between January 2008 and December 2023 with a clinical diagnosis of UC were included in the study. Among them, 456 patients had progressive disease (pN+) and underwent radical surgery and regional lymph node dissection. A total of 154 patients were excluded owing to poor paraffin samples or incomplete characteristic information. Finally, 302 patients with pathologically

confirmed advanced UC, including 179 with bladder cancer, 63 with renal pelvis cancer, 36 with ureter cancer, and 24 with urethral cancer, were included and the patient selection flow chart was shown in **Supplementary Figure 1**. The median patient age ranged from 62 years (bladder cancer) to 65.5 years (urethral cancer), and the proportion of males was greater in all subtypes (66.7%-89.3%). The detailed baseline clinical characteristics are shown in **Supplementary Table 1**.

In terms of pathology, high-grade tumors accounted for 91.6%-100.0% of the tumors across subtypes, and pure urothelial carcinoma was most common in ureter cancer (88.9%). Bladder cancer patients had the highest mean number of removed LNs (24.88 ± 12.41) and positive LNs (6.63 ± 8.50) and showed the highest prevalence (83.2%) of lymphovascular invasion (LVI) and perineural invasion (PNI). The metastasis rates varied, with renal pelvis cancer having the highest rate (14.3%) and ureter cancer having the lowest rate (2.8%). During the median follow-up time of 34.2 months, 114 (63.6%), 38 (60.3%), 26 (72.2%) and 11 (45.8%) deaths were observed among patients with bladder cancer, renal pelvis cancer, ureter cancer and urethral cancer, respectively. With respect to adjuvant treatment, 58.9% (178/302) of patients received cisplatin-based chemotherapy, and 24.2% (73/302) of patients received combined immunotherapy. Neoadjuvant therapy was rarely used. The prognosis of

advanced upper-tract urothelial carcinoma was worse than that of bladder cancer **(Supplementary Table 1)**.

Prognostic Value of ADC-Related Markers in Primary Urothelial Tumors and mLNs

To detect the expression of ADC-related markers in primary and metastatic lymph nodes in UC patients, IHC staining for HER2, HER3, Nectin4 and Trop2 was performed, and the patterns are shown in **Supplementary Figure 2**.

Furthermore, we investigated the prognostic value of ADC-related genes in advanced UC. We found that the expression of HER2, HER3, Nectin4 and Trop2 was not associated with pT, pN or LVI/PNI in advanced UC **(Supplementary Table 2-5)**. However, survival analysis revealed that high HER2 expression (2/3+) and positive HER3 and Trop2 expression (1-3+) were associated with shorter OS and PFS in patients with advanced UC **(Supplementary Table 2 and Supplementary Figure 4).** In addition, high-risk clinical and pathological factors such as advanced pN stage, metastasis, variant histology and extranodal extension were associated with poor outcomes **(Supplementary Table 3)**. We incorporated the above univariate factors into a multivariate logistic regression analysis, and the results indicated that only HER2 overexpression in mLNs was an independent prognostic ADC-related

gene factor for predicting poor OS (HR=2.20; 95% CI, 1.43-3.37; $P < 0.001$) and PFS (HR=1.62; 95% CI, 1.07-2.45; $P = 0.022$) **(Supplementary Table 3)**. Our results suggest the significant prognostic implications of these markers in tumors, especially in mLNs, which deserve more attention.

References

1. Goud KI, Dayakar S, Vijayalaxmi K, Babu SJ, Reddy PV. Evaluation of HER-2/neu status in breast cancer specimens using immunohistochemistry (IHC) & fluorescence in-situ hybridization (FISH) assay. Indian J Med Res. 2012;135(3):312-7.
2. Tumor Pathology Committee of Chinese Anti-Cancer A, Expert Committee on Urothelial Carcinoma of Chinese Society of Clinical O. [Clinical pathological expert consensus on HER-2 testing in urothelial carcinoma in China]. Zhonghua Zhong Liu Za Zhi. 2021;43(10):1001-6.
3. Tan X, Liu Z, Cai T, Wang Y, Wu Z, Qin Z, et al. Prognostic Significance of HER2 Expression in Patients with Bacillus Calmette-Guerin-exposed Non-muscle-invasive Bladder Cancer. Eur Urol Oncol. 2024;7(4):760-9.
4. Challita-Eid PM, Satpayev D, Yang P, An Z, Morrison K, Shostak Y, et al. Enfortumab Vedotin Antibody-Drug Conjugate Targeting Nectin-4 Is a Highly Potent Therapeutic Agent in Multiple Preclinical Cancer Models. Cancer Res. 2016;76(10):3003-13.
5. Tomiyama E, Fujita K, Nakano K, Kuwahara K, Minami T, Kato T, et al. Trop-2 in Upper Tract Urothelial Carcinoma. Curr Oncol. 2022;29(6):3911-21.
6. Hoffman-Censits JH, Lombardo KA, Parimi V, Kamanda S, Choi W, Hahn NM, et al. Expression of Nectin-4 in Bladder Urothelial Carcinoma, in Morphologic Variants, and Nonurothelial Histotypes. Appl Immunohistochem Mol Morphol. 2021;29(8):619-25.

Supplementary Table 1: The clinical and pathological baselines of 302 urothelial cancer patients.

**Variables (n, %)**<br>**Total cohort (*n* = 302)**	**Bladder Cancer (*n* = 179)**	**Renal Pelvis Cancer (*n* = 63)**	**Ureter Cancer (*n* = 36)**	**Urethral Cancer[b] (*n* = 24)**
**Age (years, median ± IQR)**	62 [56-68]	64 [58-69]	63 [57.25-68.75]	65.5 [60.5-75.75]
**Removed LNs (mean ± SD):**	24.88 ± 12.41	9.55 ± 8.21	10.02 ± 9.40	14.90 ± 11.05
**Positive LNs (mean ± SD):**	6.63 ± 8.50	2.63 ± 2.62	2.77 ±4.18	1.20 ± 1.40
**Sex:**				
Male	160 (89.3)	44 (69.8)	24 (66.7)	19 (79.2)
Female	19 (10.4)	19 (30.2)	12 (33.3)	5 (20.8)
**pT stage:**				
pTa	0	0	0	3 (12.5)
pT1	6 (3.4)	3 (4.8)	5 (13.9)	5 (20.8)
pT2	27 (15.1)	6 (9.5)	8 (22.2)	7 (29.2)
pT3	75 (41.9)	33 (52.4)	16 (44.4)	6 (25.0)
pT4	71 (39.6)	21 (33.3)	7 (19.4)	3 (12.5)
**pN stage:**				
pN0	0	0	0	14 (58.3)
pN1	58 (32.5)	25 (39.7)	13 (36.1)	3 (12.5)
pN2	57 (31.8)	38 (59.4)	23 (63.9)	7 (29.2)
pN3	64 (35.7)	-	-	-
**Metastasis:**	18 (10.0）	9 (14.3)	1 (2.8)	1 (4.1)
**LVI/PNI:**	149 (83.2)	32 (50.7)	21 (58.3)	6 (25.0)
**Histologic grade:**				
High-grade	164 (91.6)	58 (92.1)	38 (100.0)	22 (91.7)
Low-grade	4 (2.2)	1 (1.6)	0	1 (4.2)
Others[a]	11 (6.1)	4 (6.3)	0	1 (4.2)
**Pure urothelial carcinoma:**	108 (60.3)	43 (68.3)	32 (88.9)	15 (62.5)
**With Variant histologies:**	71 (39.6)	20 (31.7)	4 (11.1)	9 (37.5)
Squamous	38 (21.2)	15 (23.8)	2 (5.6)	8 (33.3)
Adenocarcinoma	24 (13.4)	2 (3.2)	1 (2.8)	2 (8.3)
Micropapillary	5 (2.8)	0	0	0
Sarcomatoid	7 (3.9)	5 (7.9)	1 (2.8)	0
Plasmacytoid	6 (3.4)	0	0	0
Neuroendocrine/Small-cell	6 (3.4)	0	0	0
**Treatment:**				
Neoadjuvant therapy	10 (5.4)	5 (7.9)	2 (5.6)	0
Cisplatin-based chemotherapy	110 (61.4)	39 (61.9)	21 (58.3)	8 (80.0)
ICI-based Immunotherapy	40 (22.3)	18 (28.6)	13 (36.1)	2 (20.0)
**Outcomes:**				
Recurrence	96 (53.6)	41 (65.1)	26 (72.2)	10 (41.7)

Progression	115 (64.2)	47 (74.6)	28 (77.8)	13 (54.2)
Death	114 (63.6)	38 (60.3)	26 (72.2)	11 (45.8)
mOS (95% Cl)	27.97 (19.05-36.88)	19.80 (12.34-27.27)	25.93 (17.19-34.67)	33.67 (21.86-45.48)
mPFS (95% Cl)	25.13 (15.95-34.32)	13.23 (5.52-20.95)	19.07 (8.89-29.25)	34.83 (8.02-61.65)

[a]IQR: interquartile range; [b]10 urethral cancer patients with metastatic lymph nodes were used for subsequent analysis of expression heterogeneity. LVI: lymphovascular invasion; PNI: perineural invasion; LNs: lymph nodes; SD: standard deviation; ICI: immune checkpoint inhibitor; OS: overall survival; PFS: progression-free survival

Supplementary Table 2: Survival analyzes of ADC-relative target expression in different urothelial cancers with primary tumors and mLNs

	Bladder Cancer (*n* = 179) (95% Cl)				Renal Pelvis Cancer (*n* = 63) (95% Cl)				Ureter Cancer (*n* = 36) (95% Cl)				Urethral Cancer (*n* = 10) (95% Cl)			
	mOS (mo)	*P*	mPFS (mo)	*P*	mOS (mo)	*P*	mPFS (mo)	*P*	mOS (mo)	*P*	mPFS (mo)	*P*	mOS (mo)	*P*	mPFS (mo)	*P*
**Primary Tumors**																
**HER2 expression**		**0.005**		**0.008**				**0.046**		0.399		0.972		**0.009**		**0.009**
Low (0/1+)	44.67 (23.19-66.14)		36.40 (8.02-64.78)		21.23 (NA-53.61)	0.338	14.03 (1.51-26.56)		27.50 (22.52-32.48)		19.70 (1.91-37.49)		62.33 (23.93-100.7)		35.43 (34.47-36.39)	
High (2/3+)	22.20 (14.51-29.89)		17.53 (7.20-27.87)		18.90 (12.96-24.84)		12.17 (4.72-19.61)		17.13 (12.79-21.47)		17.73 (16.45-19.02)		21.13 (NA-42.69)		9.57 (9.23-9.91)	
**HER3 expression**		**0.015**		**0.044**		0.743		0.127		0.648		0.936		**0.044**		**0.015**
Negative (0)	38.73 (20.68-56.79)		28.50 (9.85-47.15)		19.03 (13.41-24.66)		14.03 (4.78-23.28)		25.93 (13.15-38.72)		19.07 (15.12-23.01)		26.50 (11.66-41.34)		14.62 (NA-46.15)	
Positive (1-3+)	22.20 (12.84-31.56)		17.73 (1.52-33.95)		24.03 (2.50-45.47)		9.77 (2.85-16.68)		17.13 (1.06-33.21)		17.13 (3.16-31.11)		8.43 (NA-18.04)		8.43 (2.06-14.80)	
**Nectin4 expression**		0.139		0.193		0.417		0.711		0.071		0.177		0.365		0.522
Negative (0)	37.83 (10.69-64.98)		26.47 (8.89-44.05)		14.80 (6.61-23.00)		9.77 (5.98-13.56)		16.60 (4.94-28.26)		11.33 (NA-22.90)		25.97 (18.23-33.70)		10.97 (8.73-13.21)	
Positive (1-3+)	26.07 (15.76-36.38)		21.70 (10.79-32.61)		20.20 (2.20-38.20)		13.97 (6.94-21.00)		25.93 (177.9-33.93)		19.07 (15.69-22.44)		33.67 (NA-87.39)		11.67 (5.94-17.40)	
**Trop2 expression**		**0.010**		**0.016**		0.755		0.165		0.560		0.619		-		-
Negative (0)	118.23 (13.59-222.9)		68.03 (NA-147.70)		15.03 (NR)		NR		16.70 (NR)		11.33 (NR)		-		-	
Positive (1-3+)	24.33 (15.89-32.78)		17.43 (8.93-25.94)		19.80 (13.66-29.94)		12.17 (7.07-17.26)		25.53 (17.28-33.79)		17.90 (14.55-21.25)		25.97 (17.65-34.28)		-	
**Metastatic LNs**																
**HER2 expression**		**<0.001**		**<0.001**		**<0.001**		**0.001**		0.052		0.198		0.075		**0.020**
Low (0/1+)	46.23 (21.27-71.20)		37.13 (21.24-53.02)		52.93 (NR)		29.63 (8.13-51.14)		28.87 (NA-57.85)		25.93 (NA-67.80)		38.33 (11.78-64.89)		34.83 (NA-72.05)	
High (2/3+)	19.23 (12.79-25.68)		16.57 (10.19-22.94)		12.73 (7.09-18.37)		8.20 (6.03-10.37)		16.70 (8.76-24.65)		17.13 (6.67-27.60)		21.13 (NA-48.40)		9.57 (7.13-12.00)	
**HER3 expression**		**0.020**		0.060		0.580		0.699		0.423		0.494		0.920		0.856
Negative (0)	36.47 (25.32-47.62)		28.73 (11.65-45.82)		18.67 (13.57-23.76)		12.17 (3.65-20.69)		25.93 (13.15-38.72)		19.70 (4.65-34.75)		25.97 (11.85-40.09)		10.97 (6.88-15.06)	
Positive (1-3+)	18.30 (10.37-26.23)		17.43 (2.04-32.83)		21.23 (NA-49.16)		13.23 (6.46-20.00)		20.17 (9.02-31.32)		14.03 (6.02-22.04)		33.67 (NR)		11.67 (NR)	
**Nectin4 expression**		0.571		0.544		0.441		0.159		0.728		0.417		0.822		0.687
Negative (0)	30.73 (22.57-38.90)		26.47 (10.59-42.34)		20.20 (NA-54.74)		16.93 (7.99-25.87)		27.50 (12.14-42.86)		14.03 (5.74-22.32)		26.50 (25.36-27.65)		17.50 (3.47-31.53)	
Positive (1-3+)	27.97 (16.01-39.92)		19.50 (7.44-31.56)		19.80 (14.13-25.47)		9.63 (3.02-16.25)		21.67 (12.90-30.43)		19.07 (15.51-22.63)		12.73 (3.50-21.97)		9.43 (7.29-11.58)	
**Trop2 expression**		**<0.001**		**0.005**		0.930		0.739		0.592		0.735		0.422		0.636
Negative (0)	68.03 (NR)		46.90 (9.13-104.67)		19.03 (NA-54.87)		5.30 (NA-35.32)		27.50 (NA-71.14)		13.10 (NA-56.74)		21.13 (NR)		10.97 (NR)	
Positive (1-3+)	19.50 (12.58-26.42)		16.60 (10.19-23.01)		20.20 (12.81-27.59)		13.23 (5.83-20.64)		21.67 (14.98-28.36)		17.90 (14.44-21.36)		26.50 (24.94-28.06)		11.67 (5.53-17.80)	

ADC: antibody-drug conjugate; Cl: confidence interval; NA: Not available; NR: Not reached; mOS: medium overall survival; mPFS: medium progression-free survival; LNs: lymph node.

Supplementary Table 3: Survival analysis of ADC-relative target expression and clinical features in pN+ urothelial cancer patients

	Univariate Kaplan-Meier analysis				Multivariate Cox regression analysis[a]			
	OS		PFS		OS		PFS	
**Variable**	**OR (95% CI)**	***P***	**OR (95% CI)**	***P***	**HR (95% CI)**	***P***	**HR (95% CI)**	***P***
**Age (⩾62 vs. <62 years)**	1.33 (1.00-1.78)	0.050	1.22 (0.92-1.61)	0.163	-		-	
**Primary sites (vs. Bladder cancer)**	Reference		Reference		-		-	
Renal Pelvis cancer	0.96 (0.67-1.39)	0.845	1.28 (0.91-1.79)	0.157				
Ureter cancer	1.08 (0.71-1.66)	0.717	1.21 (0.80-1.83)	0.373				
Urethral Cancer	1.33 (0.67-2.62)	0.412	1.72 (0.90-3.29)	0.100				
**pT stage (vs. ⩽ pT1)**	Reference		Reference		-		-	
pT2	0.96 (0.44-2.13)	0.930	0.94 (0.45-2.00)	0.883				
pT3	1.58 (0.77-3.26)	0.216	1.64 (0.82-3.25)	0.160				
pT4	1.59 (0.76-3.31)	0.214	1.61 (0.81-3.23)	0.177				
**pN stage (vs. pN1)**	Reference		Reference		Reference		Reference	
pN2	1.71 (1.21-2.42)	**0.003**	1.79 (1.28-2.50)	**<0.001**	1.30 (0.89-1.89)	0.170	1.54 (1.07-2.21)	**0.019**
pN3	2.14 (1.44-3.18)	**<0.001**	2.32 (1.58-3.40)	**<0.001**	1.17 (0.74-1.84)	0.500	1.39 (0.89-2.16)	0.145
**Metastasis (Yes vs. No)**	2.42 (1.59-3.67)	**<0.001**	2.94 (1.96-4.41)	**<0.001**	1.64 (1.05-2.58)	**0.031**	1.99 (1.27-3.14)	**0.003**
**Variant histologies (Yes vs. No)**	1.64 (1.22-2.20)	**0.001**	1.57 (1.18-2.09)	**0.002**	1.53 (1.13-2.07)	**0.006**	1.43 (1.06-1.92)	**0.017**
**LVI/PNI (Yes vs. No)**	1.47 (1.06-2.05)	**0.022**	1.22 (0.90-1.67)	0.198	1.17 (0.82-1.65)	0.386	-	
**Extranodal extension (Yes vs. No)**	1.81 (1.33-2.46)	**<0.001**	1.76 (1.30-2.38)	**<0.001**	1.47 (1.06-2.06)	**0.022**	1.32 (0.95-1.83)	0.093
**Primary tumor expression**								
HER2 (High vs. Low)	1.65 (1.23-2.20)	**<0.001**	1.59 (1.20-2.11)	**0.001**	0.82 (0.53-1.25)	0.353	1.01 (0.67-1.52)	0.971
HER3 (Positive vs. Negative)	1.48 (1.11-1.97)	**0.008**	1.39 (1.05-1.84)	**0.022**	1.27 (0.89-1.82)	0.194	1.28 (0.95-1.71)	0.104
Nectin4 (Positive vs. Negative)	1.09 (0.77-1.54)	0.624	1.11 (0.79-1.56)	0.538	-		-	
Trop2 (Positive vs. Negative)	1.76 (1.10-2.80)	**0.018**	2.04 (1.27-3.27)	**0.003**	1.47 (0.88-2.46)	0.137	1.79 (1.07-3.00)	**0.027**
**Metastatic LNs expression**								
HER2 (High vs. Low)	2.32 (1.73-3.12)	**<0.001**	1.95 (1.46-2.59)	**<0.001**	2.20 (1.43-3.37)	**<0.001**	1.62 (1.07-2.45)	**0.022**
HER3 (Positive vs. Negative)	1.35 (1.01-1.82)	0.050	1.28 (0.96-1.71)	0.094	-		-	
Nectin4 (Positive vs. Negative)	1.17 (0.86-1.60)	0.310	1.12 (0.83-1.51)	0.448	-		-	
Trop2 (Positive vs. Negative)	1.79 (1.23-2.62)	**0.002**	1.64 (1.15-2.35)	**0.007**	1.29 (0.84-1.99)	0.247	1.11 (0.74-1.66)	0.607

[a]The clinical features were subjected to univariate Kaplan-Meier survival analysis, and the significant factors were evaluated by following multivariate Cox regression analysis with forward method.

ADC: antibody-drug conjugate; LVI: lymphovascular invasion; PNI: perineural invasion; LNs: lymph nodes; NA:Not available; OS: overall survival; PFS: progression-free survival.

Supplementary Table 4: The association between ADC-relative genes expression in primary bladder, renal pelvis tumors and clinical features

Bladder Cancer (*n*=179)

	HER2 expression			HER3 expression			Nectin4 expression			Trop2 expression		
	low	high	*P*	Neg.	Pos.	*P*	Neg.	Pos.	*P*	Neg.	Pos.	*P*
**Total (%)**	78 (43.6)	101 (56.4)		86 (48.0)	93 (52.0)		40 (22.3)	139 (77.7)		30 (16.8)	149 (83.2)	
**Age**			0.618			0.607			0.904			0.133
≤ 62	38 (21.2)	53 (29.6)		42 (23.5)	49 (27.4)		20 (11.2)	71 (39.7)		19 (10.6)	72 (40.2)	
> 62	40 (22.3)	48 (26.8)		44 (24.6)	44 (24.6)		20 (11.2)	68 (38.0)		11 (6.1)	77 (43.0)	
**pT stage**			0.223[a]			0.737[a]			0.009[a]			0.566[a]
pT1	2 (1.1)	4 (2.2)		4 (2.2)	2 (1.1)		2 (1.1)	4 (2.2)		2 (1.1)	4 (2.2)	
pT2	16 (8.9)	11 (6.1)		14 (7.8)	13 (7.3)		5 (2.8)	22 (12.3)		5 (2.8)	22 (12.3)	
pT3	34 (19.0)	41 (22.9)		36 (20.1)	39 (21.8)		25 (14.0)	50 (27.9)		13 (7.3)	62 (34.1)	
pT4	26 (14.5)	45 (25.1)		32 (17.9)	39 (21.8)		8 (4.5)	63 (35.2)		10 (5.6)	61 (34.1)	
**pN stage**			0.472			0.689			0.149			0.183
pN0	-	-		-	-		-	-		-	-	
pN1	27 (15.1)	31 (17.3)		29 (16.2)	29 (16.2)		18 (10.1)	40 (22.3)		14 (7.8)	44 (24.6)	
pN2	27 (15.1)	30 (16.8)		29 (16.2)	28 (15.6)		11 (6.1)	46 (25.7)		8 (4.5)	49 (27.4)	
pN3	24 (13.4)	40 (22.3)		28 (15.6)	36 (20.1)		11 (6.1)	53 (29.6)		8 (4.5)	56 (31.3)	
**LVI/PNI**			0.237			0.525			0.534			0.111
No	16 (8.9)	14 (7.8)		16 (8.9)	14 (7.8)		8 (4.5)	22 (12.3)		8 (4.5)	22 (12.3)	
Yes	62 (34.6)	87 (48.6)		70 (39.1)	79 (44.1)		32 (17.9)	117 (65.4)		22 (12.3)	127 (70.9)	
**VH**			0.346			0.234			0.434			0.390
No	44 (24.6)	64 (35.8)		48 (26.8)	60 (33.5)		22 (12.3)	86 (48.0)		16 (8.9)	92 (51.4)	
Yes	34 (19.0)	37 (20.7)		38 (21.2)	33 (18.4)		18 (10.1)	53 (29.6)		14 (7.8)	57 (31.8)	

Renal Pelvis Cancer (*n* = 63)

	HER2 expression			HER3 expression			Nectin4 expression			Trop2 expression		
	low	high	*P*	Neg.	Pos.	*P*	Neg.	Pos.	*P*	Neg.	Pos.	*P*
**Total (%)**	41 (65.1)	22 (34.9)		45 (71.4)	18 (28.6)		16 (25.4)	47 (74.6)		6 (9.5)	57 (90.5)	
**Age**			0.801			0.750			0.424			0.412[a]
≤ 62	21 (33.3)	12 (19.0)		23 (36.5)	10 (15.9)		7 (11.1)	26 (41.3)		2 (3.2)	31 (49.2)	
> 62	20 (31.7)	10 (15.9)		22 (34.9)	8 (12.7)		9 (14.3)	21 (33.3)		4 (6.3)	26 (41.3)	
**pT stage**			0.463[a]			0.104[a]			0.890[a]			0.590[a]
pT1	3 (4.8)	0 (0)		3 (4.8)	0 (0)		0 (0)	3 (4.8)		0 (0)	3 (4.8)	
pT2	3 (4.8)	3 (4.8)		2 (3.2)	4 (6.3)		1 (1.6)	5 (7.9)		0 (0)	6 (9.5)	
pT3	20 (31.7)	13 (20.6)		23 (36.5)	10 (15.9)		9 (14.3)	24 (38.1)		5 (7.9)	28 (44.4)	
pT4	15 (23.8)	6 (9.5)		17 (27.0)	4 (6.3)		6 (9.5)	15 (23.8)		1 (1.6)	20 (31.7)	
**pN stage**			0.350			0.935			0.700			0.389[a]
pN0	-	-		-	-		-	-		-	-	
pN1	18 (28.6)	7 (11.1)		18 (28.6)	7 (11.1)		7 (11.1)	18 (28.6)		1 (1.6)	24 (38.1)	
pN2	23 (26.5)	15 (23.8)		27 (42.9)	11 (17.5)		9 (14.3)	29 (46.0)		5 (7.9)	33 (52.4)	
pN3	-	-		-	-		-	-		6 (9.5)	57 (90.5)	
**LVI/PNI**			0.926			0.936			0.514			0.672[a]
No	20 (31.7)	11 (17.5)		22 (34.9)	9 (14.3)		9 (14.3)	22 (34.9)		2 (3.2)	29 (46.0)	
Yes	21 (33.3)	11 (17.5)		23 (36.5)	9 (14.3)		7 (11.1)	25 (39.7)		4 (6.3)	28 (44.4)	
**VH**			0.576			0.864			0.069			1.000[a]
No	27 (42.9)	16 (25.4)		31 (49.2)	12 (19.0)		8 (12.7)	35 (55.6)		4 (6.3)	39 (61.9)	
Yes	14 (22.2)	6 (9.5)		14 (22.2)	6 (9.5)		8 (12.7)	12 (19.0)		2 (3.2)	18 (28.6)	

[a]Fisher's exact test; LVI: lymphovascular invasion; PNI: perineural invasion; VH: Variant histologies.

Supplementary Table 5: The association between ADC-relative genes expression in primary ureter, urethral tumors and clinical features

	Ureter Cancer (*n*=36)											
	**HER2 expression**			**HER3 expression**			**Nectin4 expression**			**Trop2 expression**		
	**low**	**high**	***P***	**Neg.**	**Pos.**	***P***	**Neg.**	**Pos.**	***P***	**Neg.**	**Pos.**	***P***
**Total (%)**	21 (58.3)	15 (41.7)	0.476	21 (58.3)	15 (41.7)		4 (11.1)	32 (88.9)		2 (5.6)	34 (94.4)	
**Age**			0.418			0.908			0.277[a]			0.144[a]
≤ 62	7 (19.4)	7 (19.4)		8 (22.2)	6 (16.7)		3 (8.3)	11 (30.6)		2 (5.6)	12 (33.3)	
> 62	14 (38.9)	8 (22.2)		13 (36.1)	9 (25.0)		1 (2.8)	21 (58.3)		0 (0)	22 (61.1)	
**pT stage**			0.472[a]			0.596[a]			0.804[a]			0.213[a]
pT1	4 (11.1)	1 (2.8)		4 (11.1)	1 (2.8)		1 (2.8)	4 (11.1)		1 (2.8)	4 (11.1)	
pT2	5 (13.9)	3 (8.3)		4 (11.1)	4 (11.1)		1 (2.8)	7 (19.4)		1 (2.8)	7 (19.4)	
pT3	7 (19.4)	9 (25.0)		10 (27.8)	6 (16.7)		1 (2.8)	15 (41.7)		0 (0)	16 (44.4)	
pT4	5 (13.9)	2 (5.6)		3 (8.3)	4 (11.1)		1 (2.8)	6 (16.7)		0 (0)	7 (19.4)	
**pN stage**			1.000[a]			0.681			0.274[a]			0.525[a]
pN0	-	-		-	-		-	-		-	-	
pN1	8 (22.2)	5 (13.9)		7 (19.4)	6 (16.7)		0 (0)	13 (36.1)		0 (0)	13 (36.1)	
pN2	13 (36.1)	10 (27.8)		14 (38.9)	9 (25.0)		4 (11.1)	19 (52.8)		2 (5.6)	21 (58.3)	
pN3	-	-		-	-		-	-		-	-	
**LVI/PNI**			1.000			0.391[a]			0.626[a]			1.000[a]
No	9 (25.0)	6 (16.7)		10 (27.8)	5 (13.9)		1 (2.8)	14 (38.9)		1 (2.8)	14 (38.9)	
Yes	12 (33.3)	9 (25.0)		11 (30.6)	10 (27.8)		3 (8.3)	18 (50.0)		1 (2.8)	20 (55.6)	
**VH**			0.125[a]			0.626[a]			0.390[a]			1.000[a]
No	17 (47.2)	15 (41.7)		18 (50.0)	14 (38.9)		3 (8.3)	29 (80.6)		2 (5.6)	30 (83.3)	
Yes	4 (11.1)	0 (0)		3 (8.3)	1 (2.8)		1 (2.8)	3 (8.3)		0 (0)	4 (11.1)	

	Urethral Cancer (*n* = 24)											
	**HER2 expression**			**HER3 expression**			**Nectin4 expression**			**Trop2 expression**		
	**low**	**high**	***P***	**Neg.**	**Pos.**	***P***	**Neg.**	**Pos.**	***P***	**Neg.**	**Pos.**	***P***
**Total (%)**	12 (50.0)	12 (50.0)		8 (33.3)	16 (66.7)		3 (12.5)	21 (87.5)		1 (4.2)	23 (95.8)	
**Age**			0.667[a]			1.000[a]			0.249[a]			1.000[a]
≤ 62	5 (20.8)	3 (12.5)		3 (12.5)	5 (20.8)		2 (8.3)	6 (25.0)		0 (0)	8 (33.3)	
> 62	7 (29.2)	9 (37.5)		5 (20.8)	11 (45.8)		1 (4.2)	15 (62.5)		1 (4.2)	15 (62.5)	
**pT stage**			0.723[a]			0.409[a]			0.417[a]			0.375[a]
pT1	5 (20.8)	3 (12.5)		1 (4.2)	7 (29.2)		0 (0)	8 (33.3)		0 (0)	8 (33.3)	
pT2	4 (16.7)	3 (12.5)		3 (12.5)	4 (16.7)		1 (4.2)	6 (25.0)		0 (0)	7 (29.2)	
pT3	2 (22.3)	4 (16.7)		3 (12.5)	3 (12.5)		1 (4.2)	5 (20.8)		1 (4.2)	5 (20.8)	
pT4	1 (4.2)	2 (8.3)		1 (4.2)	2 (8.3)		1 (4.2)	2 (8.3)		0 (0)	3 (12.5)	
**pN stage**			0.360[a]			0.022[a]			0.028[a]			1.000[a]
pN0	9 (37.5)	5 (20.8)		2 (8.3)	12 (50.0)		0 (0)	14 (58.3)		1 (4.2)	13 (36.1)	
pN1	1 (4.2)	2 (8.3)		1 (4.2)	2 (8.3)		0 (0)	3 (12.5)		0 (0)	3 (12.5)	
pN2	2 (8.3)	5 (20.8)		5 (20.8)	2 (8.3)		3 (12.5)	4 (16.7)		0 (0)	7 (29.2)	
pN3	-	-		-	-		-	-		-	-	
**LVI/PNI**			1.000[a]			0.129[a]			0.143[a]			0.250[a]
No	9 (37.5)	9 (37.5)		4 (16.7)	14 (58.3)		1 (4.2)	17 (70.8)		0 (0)	18 (75.0)	
Yes	3 (21.5)	3 (21.5)		4 (16.7)	2 (8.3)		2 (8.3)	4 (16.7)		1 (4.2)	5 (20.8)	
**VH**			0.400[a]			0.412[a]			1.000[a]			0.375[a]
No	6 (25.0)	9 (37.5)		4 (16.7)	11 (45.8)		2 (8.3)	13 (54.2)		0 (0)	15 (62.5)	
Yes	6 (25.0)	3 (12.5)		4 (16.7)	5 (20.8)		1 (4.2)	8 (33.3)		1 (4.2)	8 (33.3)	

[a]Fisher's exact test; [b]Urethral Cancer pT1 including three pTa and five pT1 patients; LVI: lymphovascular invasion; PNI: perineural invasion; VH: Variant histologies

Supplementary Table 6: Survival analysis of ADC-relative targets heterogeneous expression in primary urothelial tumors and mLNs.

	IHC expression score consistency						Clinical expressed stratification consistency				
	N	mOS (mo)	***P***	mPFS (mo)	***P***		*N*	mOS (mo)	***P***	mPFS (mo)	***P***
**HER2 expression**			0.595		0.435	**HER2 expression**			**0.033**		0.186
Primary = mLNs	207	26.47 (20.20-33.67)		17.90 (14.80-28.50)		Consistency in stratification	245	25.93 (19.70-32.03)		17.53 (13.80-26.07)	
Primary > mLNs	53	25.97 (14.67-72.73)		16.43 (8.13-41.97)		Primary high, mLNs low	28	68.03 (25.97-NA)		35.50 (17.50-72.73)	
Primary < mLNs	28	19.23 (14.70-NA)		14.17 (5.97-NA)		Primary low, mLNs high	15	15.13 (11.63-NA)		10.13 (5.93-NA)	
**HER3 expression**			0.086		0.261	**HER3 expression**			0.655		0.719
Primary = mLNs	207	26.57 (19.80-38.33)		19.03 (16.43-29.33)		Consistency in stratification	229	26.07 (19.70-36.30)		17.90 (15.97-28.90)	
Primary > mLNs	61	17.13 (12.13-33.67)		10.33 (8.43-31.97)		Primary positive, mLNs negative	44	24.03 (12.73-46.63)		10.67 (9.43-63.47)	
Primary < mLNs	20	25.13 (17.43-NA)		16.60 (10.30-NA)		Primary negative, mLNs positive	15	23.18 (16.70-NA)		14.23 (10.13-NA)	
**Nectin4 expression**			0.405		0.508	**Nectin4 expression**			0.438		0.794
Primary = mLNs	146	21.53 (16.70-31.97)		16.43 (10.30-28.50)		Consistency in stratification	218	24.03 (19.03-31.97)		17.50 (13.23-26.47)	
Primary > mLNs	86	34.67 (19.80-59.20)		26.80 (14.80-44.67)		Primary positive, mLNs negative	51	36.47 (19.80-NA)		25.13 (13.10-46.93)	
Primary < mLNs	56	24.33 (18.90-49.20)		17.73 (11.13-35.97)		Primary negative, mLNs positive	19	49.10 (16.60-NA)		16.60 (11.13-NA)	
**Trop2 expression**			0.059		0.161	**Trop2 expression**			0.055		0.073
Primary = mLNs	188	24.03 (18.67-31.30)		17.50 (12.33-25.93)		Consistency in stratification	240	22.20 (18.90-29.43)		16.93 (13.80-21.70)	
Primary > mLNs	65	43.80 (24.33-NA)		31.33 (14.17-NA)		Primary positive, mLNs negative	39	49.33 (27.50-NA)		31.33 (10.97-NA)	
Primary < mLNs	35	19.23 (16.70-NA)		16.93 (0.63-NA)		Primary negative, mLNs positive	9	118.23 (19.23-NA)		107.0 (34.30-NA)	

ADC: antibody-drug conjugate; Cl: confidence interval; NA: Not available; NR: Not reached; mOS: medium overall survival; mPFS: medium progression-free survival; LNs: lymph nodes.

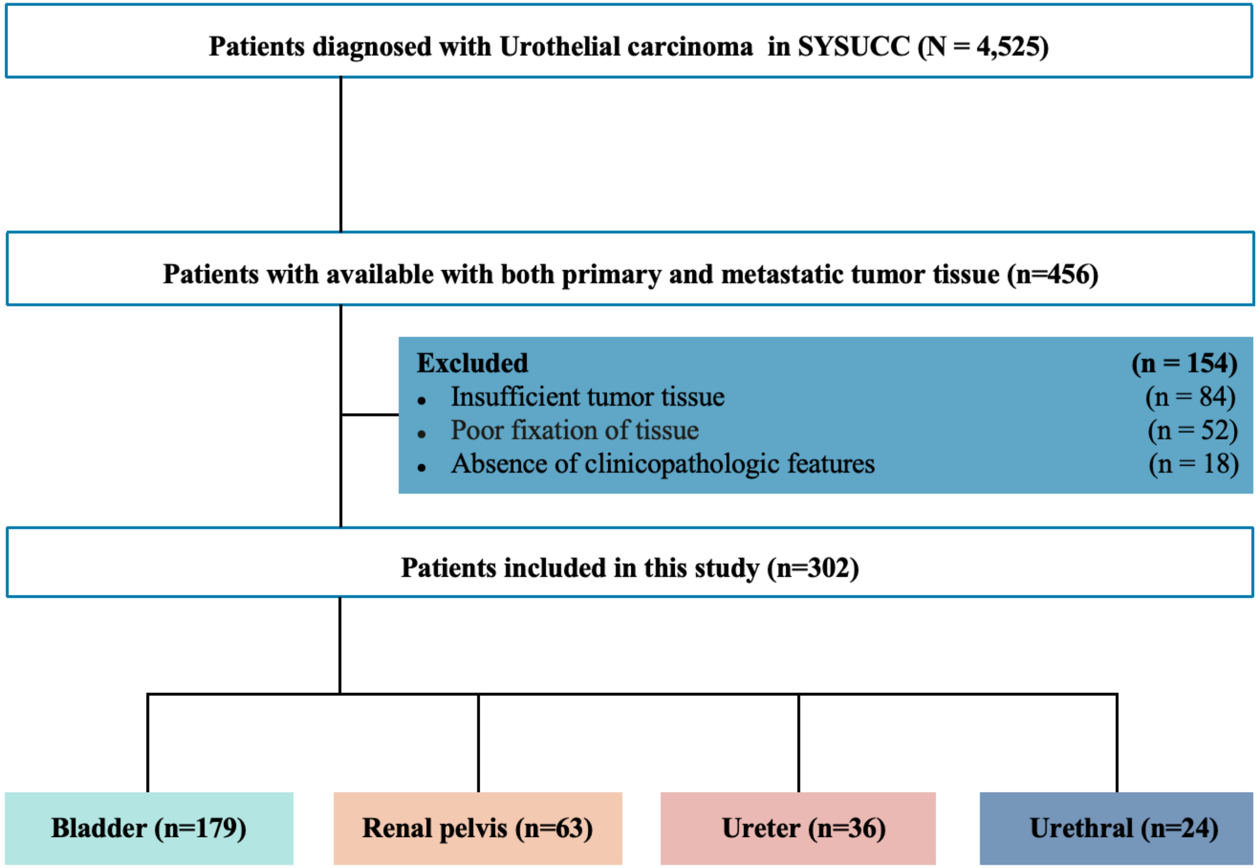

Supplementary Figure 1: Patient selection flow chart.

Supplementary Figure 2: The number of uroepithelial carcinoma patients with different ADC-related targets expression

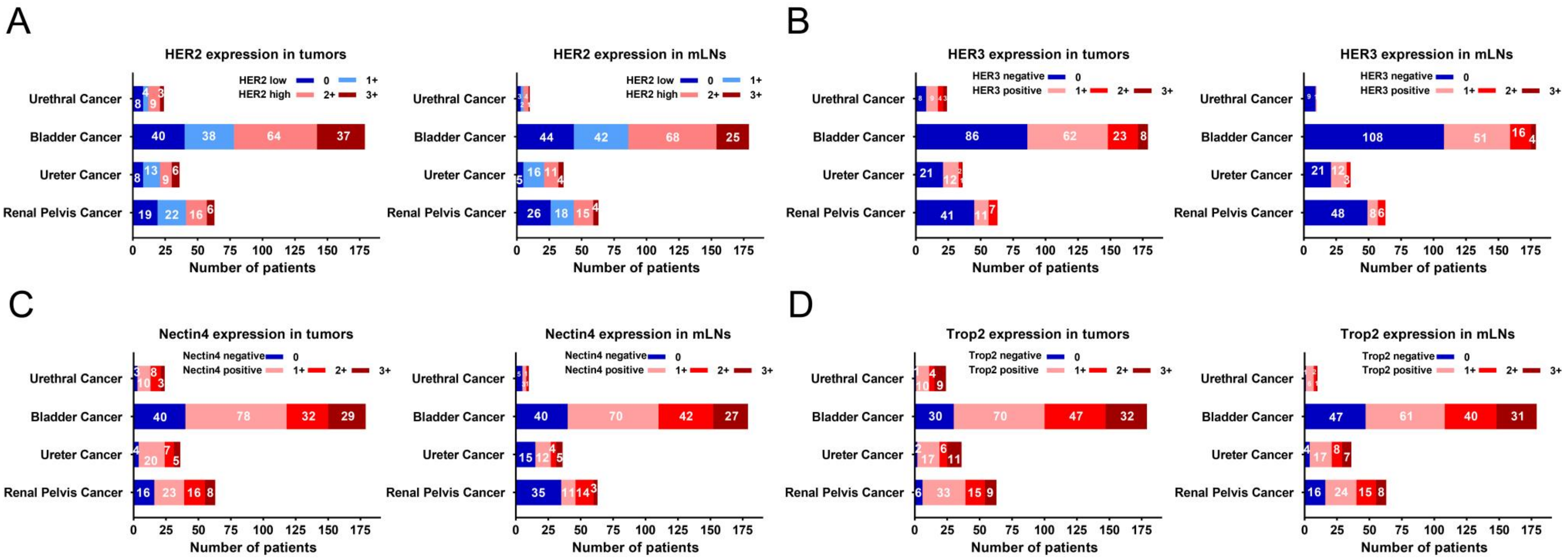

Primary tumors and metastatic lymph nodes in pairs from 179, 63 and 36 pN+ bladder cancer, renal pelvis cancer and ureter cancer were analyzed. Primary tumors from 24 patients with urothelial carcinoma and 10 metastatic nodes in pairs were also retrieved for immunohistochemical staining. ADC: antibody-drug conjugate; mLNs: metastatic lymph nodes.

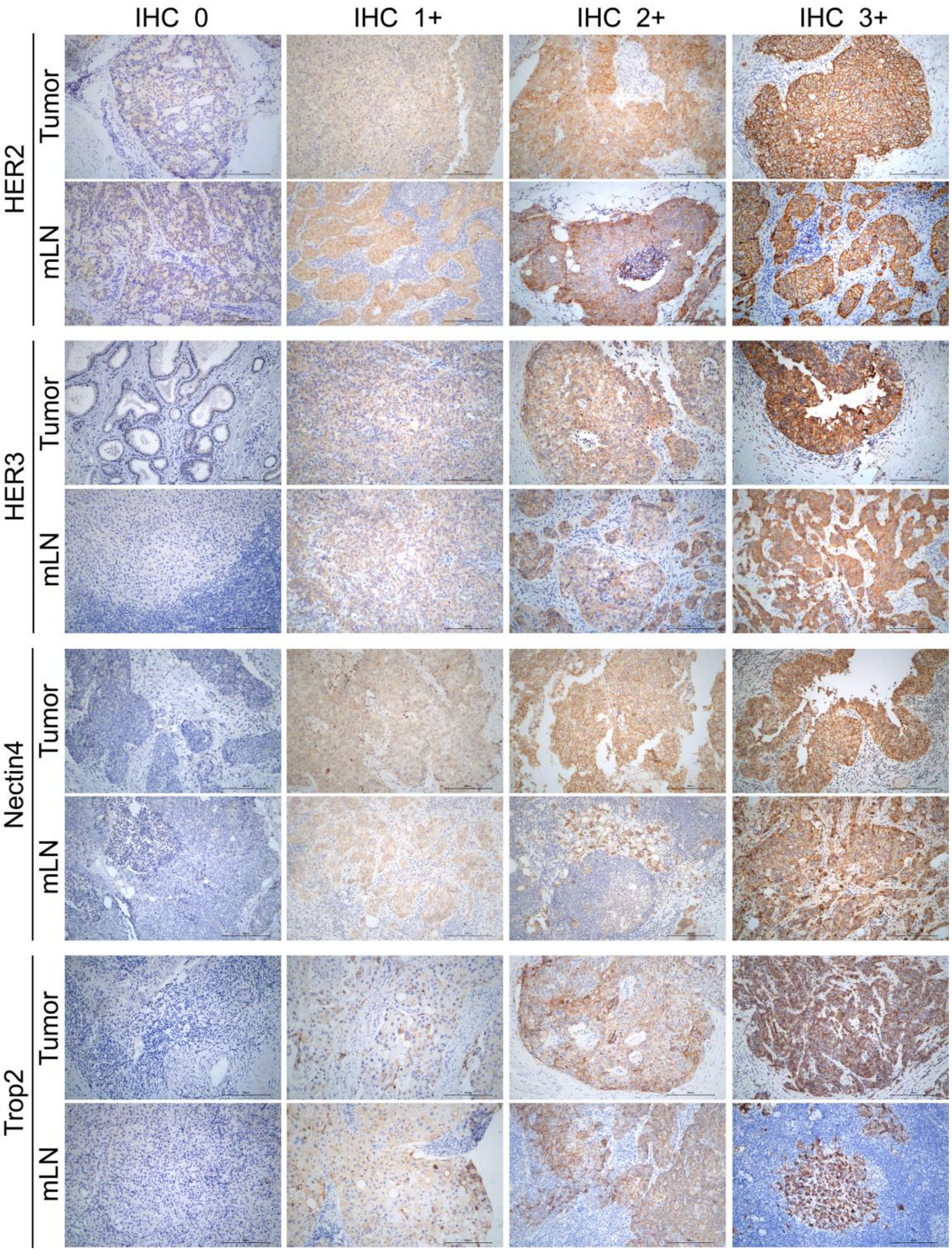

Supplementary Figure 3: Immunohistochemical staining of HER2, HER3, Nectin4 and Trop2 in urothelial tumors and paired metastatic nodes.

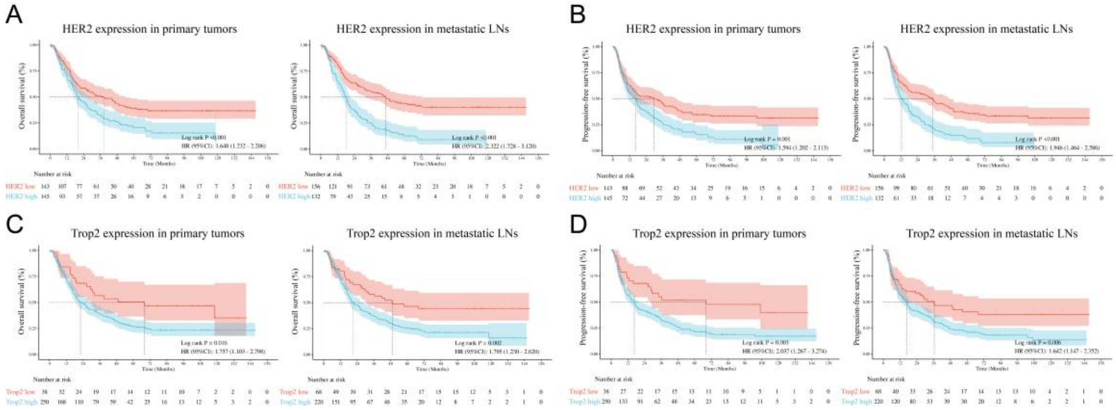

Supplementary Figure 4: HER2, Trop2 over-expression in urothelial tumors and mLNs were both associated with poor outcomes.

(A-B) HER2 high expression in primary urothelial tumors were both associated with shorter OS and PFS. (C-D) Trop2 positive expression in primary urothelial tumors were both associated with shorter OS and PFS. mLNs: metastatic lymph nodes; OS: overall survival; PFS: progression-free survival.

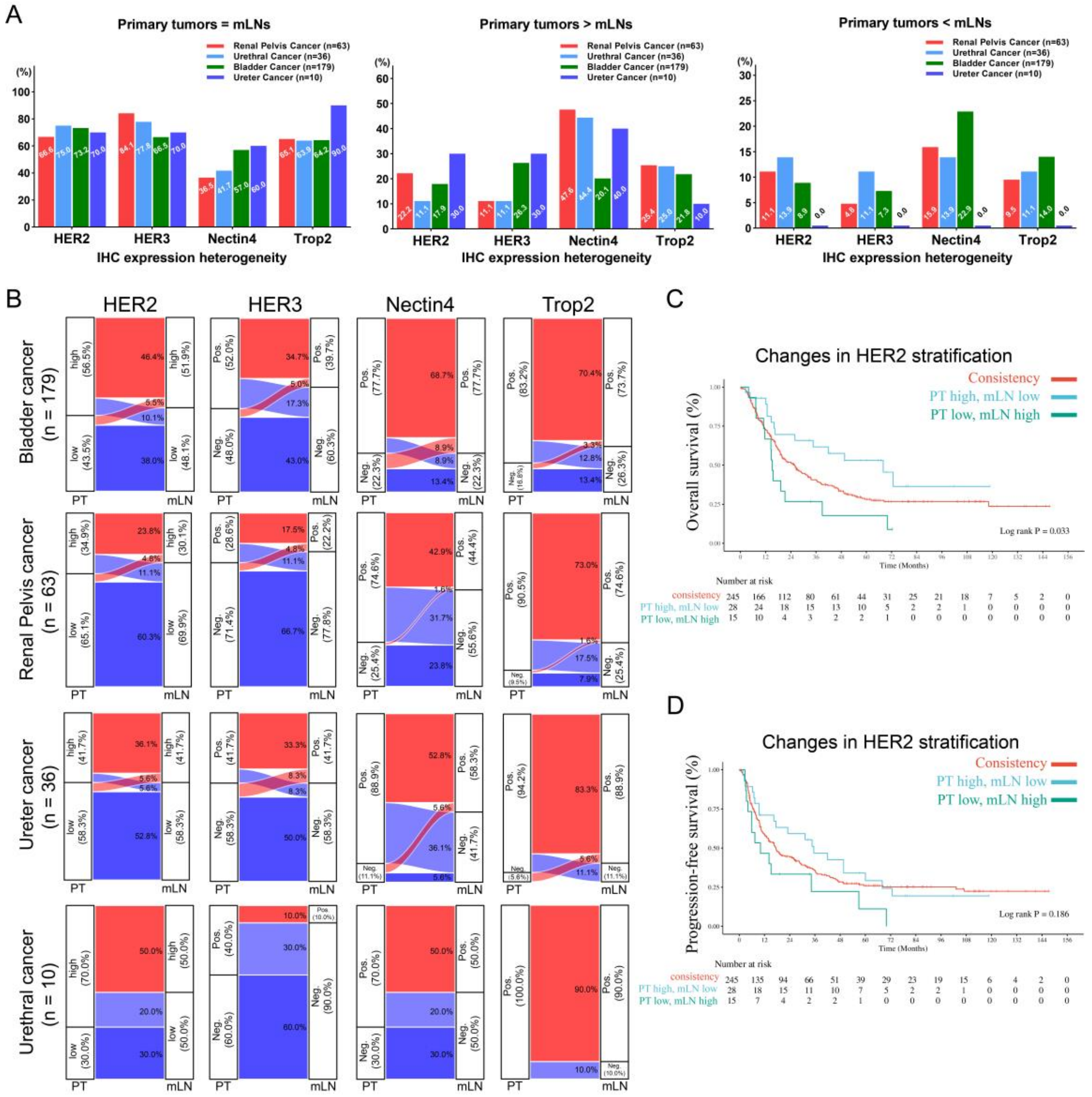

Supplementary Figure 5: Prognosis and Expression Heterogeneity of ADC-Related Markers in Primary Urothelial Tumors and mLNs.

(A) The expression heterogeneity of ADC-relative markers in primary urothelial tumors and mLNs. (B) Expression heterogeneity of ADC-related markers in primary urothelial tumors and mLNs. (C) Survival outcomes in cohorts with heterogeneous HER2 expression. mLNs: metastatic lymph nodes; OS: overall survival; PFS: progression-free survival.
